# Molecular and Enzymatic Features of Homoserine Dehydrogenase from *Bacillus subtilis*

**DOI:** 10.4014/jmb.2004.04060

**Published:** 2020-09-28

**Authors:** Do Hyeon Kim, Quyet Thang Nguyen, Gyeong Soo Ko,, Jin Kuk Yang

**Affiliations:** 1 Department of Chemistry, College of Natural Sciences, Soongsil University, Seoul 06978, Republic of Korea; 2 Department of Information Communication, Materials, and Chemistry Convergence Technology, Soongsil University, Seoul 06978, Republic of Korea

**Keywords:** Homoserine dehydrogenase, *Bacillus subtilis*

## Abstract

Homoserine dehydrogenase (HSD) catalyzes the reversible conversion of _L_-aspartate-4- semialdehyde to _L_-homoserine in the aspartate pathway for the biosynthesis of lysine, methionine, threonine, and isoleucine. HSD has attracted great attention for medical and industrial purposes due to its recognized application in the development of pesticides and is being utilized in the large scale production of _L_-lysine. In this study, HSD from *Bacillus subtilis* (BsHSD) was overexpressed in *Escherichia coli* and purified to homogeneity for biochemical characterization. We examined the enzymatic activity of BsHSD for _L_-homoserine oxidation and found that BsHSD exclusively prefers NADP^+^ to NAD^+^ and that its activity was maximal at pH 9.0 and in the presence of 0.4 M NaCl. By kinetic analysis, *K*_m_ values for _L_-homoserine and NADP^+^ were found to be 35.08 ± 2.91 mM and 0.39 ± 0.05 mM, respectively, and the *V*_max_ values were 2.72 ± 0.06 μmol/min^-1^ mg^-1^ and 2.79 ± 0.11 μmol/min^-1^ mg^-1^, respectively. The apparent molecular mass determined with size-exclusion chromatography indicated that BsHSD forms a tetramer, in contrast to the previously reported dimeric HSDs from other organisms. This novel oligomeric assembly can be attributed to the additional C-terminal ACT domain of BsHSD. Thermal denaturation monitoring by circular dichroism spectroscopy was used to determine its melting temperature, which was 54.8°C. The molecular and biochemical features of BsHSD revealed in this study may lay the foundation for future studies on amino acid metabolism and its application for industrial and medical purposes.

## Introduction

**Fig. 1:**
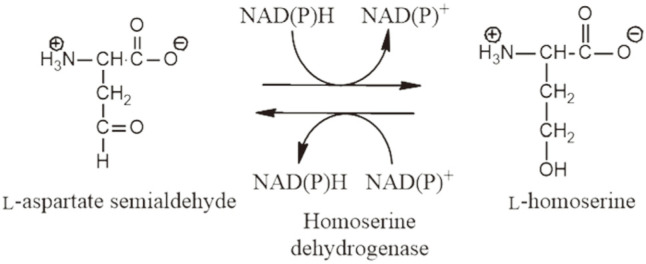
Reaction catalyzed by homoserine dehydrogenase (HSD). HSD catalyzes the reversible conversion between _L_-aspartate semialdehyde and _L_-homoserine with a cofactor NAD(P)^+^ in oxidation and or NAD(P)H in reduction.

Homoserine dehydrogenase (HSD; E.C. 1.1.1.3) catalyzes the reversible conversion of _L_-aspartate-β-semialdehyde (L-ASA) to _L_-homoserine (L-HSE) (Fig. 1). The oxidation of L-ASA to L-HSE is an important step in the aspartate pathway involved in the biosynthesis of L-threonine, L-methionine, and L-isoleucine [1]. L-ASA is the precursor for the synthesis of L-lysine via an alternative branch of the aspartate pathway. Thus, for large-scale industrial production of L-lysine, proper control of HSD is critical in improving productivity [2]. In addition to its industrial application, HSD attracts great medical interest because the aspartate pathway is present only in plants and microorganisms, not in mammals, and furthermore, HSD is a major regulatory enzyme in the pathway. This suggests that HSD is an attractive target for the development of new pesticides and antibiotics with minimal side effects in humans [2]. In this regard, the antifungal natural product (S)-2-amino-4-oxo-5-hydroxylpentanoic acid has been demonstrated to directly target HSD [1].

To date, structural and enzymatic characterization studies of HSDs have been reported for several microorganisms [2-6]. These studies have revealed features including the dimeric assembly, overall structure, catalytic key residues, and possible reaction mechanism. In particular, the dimeric assembly is a common feature for all the previously reported HSDs. For example, HSD from *Staphylococcus aureus* forms a dimer in solution as shown by size-exclusion chromatography analysis [5], and the dimeric assembly was confirmed in the crystal structure analysis. HSDs from *Saccharomyces cerevisiae*, *Thermus thermophilus*, *Pyrococcus horikoshii*, and *Sulfolobus tokodaii* also form dimers in the same manner in the crystal structure lattice [2-4,6]. All of these HSDs commonly consist of two domains: a nucleotide binding domain and a substrate binding domain. In these structures, the HSDs have a conserved dimeric interface contributed by both domains.

HSD from *Bacillus subtilis* (BsHSD) has 433 amino acid residues and includes a C-terminal ACT domain as a third domain which is not present in the above previously reported two-domain HSDs. More interestingly, we observed that BsHSD exists as a tetramer in contrast to the canonical two-domain HSDs forming dimers. Thus, to form the tetramer, BsHSD may need another type of dimeric interaction in addition to the previously characterized conserved one. Notably, the ACT domain found in many other ACT domain-containing enzymes can homo-dimerize as shown in phosphoglycerate dehydrogenase (PGDH), aspartate kinase (AK), acetohydroxyacid synthase (AHAS) and threonine deaminase (TD), which are all involved in amino acid biosynthesis processes [7-12]. In the crystal structures of these ACT domain-containing enzymes, 2 ACT domains forms a single eight-stranded sheet from the 2 four-stranded β-sheets. Thus, BsHSD represents a new class of HSD with features of the three-domain architecture and the tetramer formation. In this study, we investigated this novel BsHSD for its molecular and enzymatic features. We overexpressed the recombinant BsHSD in an *E. coli* system, and purified it to homogeneity. Then, we carried out the enzyme assay and determined the kinetic parameters for L-HSE, the substrate, and for NADP^+^, the specific cofactor. In addition, we also performed several molecular analyses including size-exclusion chromatography, circular dichroism spectroscopy, and thermal denaturation. In particular, the size-exclusion chromatography revealed that BsHSD forms a tetramer in solution. Finally, we built a homology model for its three-dimensional structure which showed the three-domain architecture of BsHSD, in contrast with the previously reported two-domain dimeric HSDs from other species [2-6]. Here, we report the detailed results from our investigations on the molecular and enzymatic features of BsHSD.

## Materials and Methods

### Construction of Expression Plasmid

The BsHSD-encoding gene, hom, was amplified using the genomic DNA of the *B. subtilis* strain 168 as a template. The following primers were used for its amplification.

5'-agatatacatatgaaagcgattcgtgta-3`(forward)

5'-ctgttctcgaggctccaaccgttcccttc-3`(reverse)

The underlined parts of the sequences indicate NdeI and Xho1 restriction enzyme sites. The expression vector, pBsHSD-26b, encoding BsHSD with an additional hexahistidine tag at its C-terminus was constructed by ligation of the digested PCR product into pET26b (Novagen, USA). The sequence of the inserted gene was confirmed by nucleotide sequencing analysis (Bionics, Korea). All reagents were purchased from Sigma-Aldrich (USA) and BioShop (Canada). Genomic DNA of *B. subtilis* ATCC 23857 was purchased from American Type Culture Collection (ATCC, USA). PCR kits and Mini Plasmid kits from GeneAll (Korea) were used for DNA purification and plasmid isolation. DNA information was retrieved from the NCBI GenBank database (https://www.ncbi.nlm.nih.gov/gnebank/). The gene ID of hom reported in this paper is CAB15216.1, and the genome accession number of *B. subtilis* strain 168 is AL009126. The protein information was retrieved from the UniProt database (https://www.uniprot.org) with the accession number P19582.

### Protein Overexpression and Purification

The expression vector, pBsHSD-26b, was transformed into Rosetta2 (DE3) strain of *E. coli* for overexpression. The cells were cultured in LB media at 37°C with shaking until the OD_600_ reached 0.5-0.8. Then, the culturing flask was cooled in an ice bath for about 10 min and isopropyl-β-D-1-thiogalactopyranoside (IPTG) was added to a final concentration of 0.5 mM to induce BsHSD expression. The cells were cultured again for an additional 20 h at 25°C. The cultured cells were harvested by centrifugation at 1,590 g for 20 min (Hanil Supra 22K), and then the cell pellets were resuspended in lysis buffer containing 20 mM Tris-HCl pH 8.0, 0.4 M NaCl, 0.1 mM TCEP, 5%glycerol, 10 mM imidazole and 0.1 mM phenyl methyl sulfonyl fluoride (PMSF). The resuspended cells were lysed by sonification and then centrifuged at 24,650 g for 60 min (Hanil Supra 22K). Next, the BsHSD was purified from the supernatants through 3 serial applications of column chromatography using a HisTrapFF, HiPrep 26/10 Desalting and Superdex-200 (GE Healthcare, USA). The protein purity was examined with Sodium Dodecyl Sulfate-Polyacrylamide Gel Electrophoresis (SDS-PAGE), and the concentration was measured using a NanoDrop 1000 (Thermo Scientific, USA).

### Activity Assay and Kinetic Analysis

The homoserine oxidation was monitored at 25°C for all enzyme assays. The absorbance at 340 nm was monitored using an Ultrospec 8000 UV/Vis spectrophotometer (GE Healthcare) and the concentration of NADPH was calculated from its extinction coefficient at 6.22 mM^-1^ cm^-1^. To determine the optimal NaCl concentration, 6 varying NaCl concentrations (50, 100, 200, 400, 800, and 1,600 mM) were examined in the presence of 100 mM CHES pH 9.5, 50 mM L-HSE, 0.5 mM NADP^+^ and 1 μM BsHSD. To screen for the optimum pH range, three different buffer systems were used to cover the inspected pH range of 7.0 to 10.0: Bis-Tris buffer for pH 7.0, Tris-HCl buffer for pH 8.0 and 9.0, CAPS buffer for pH 10.0 and 11.0. The reactions at different pHs were monitored under the standard conditions of 100 mM L-HSE, 1 mM NADP^+^, 400 mM NaCl and 0.5 μM BsHSD. To investigate the temperature dependence of the enzyme activity, the enzyme activity was measured at various temperatures from 25oC to 50oC with increments of 5oC in a reaction mixture containing 100 mM L-HSE, 1 mM NADP^+^, 400 mM NaCl, 0.5 μM BsHSD and 100 mM CHES buffer, at pH 9.0. Subsequent kinetic analyses were carried out under the determined optimum condition for NaCl concentration and pH (400 mM NaCl and pH 9.0) for 0.5 μΜ BsHSD. The cofactor preference between 2 mM NAD^+^ and 1 mM NADP^+^ was investigated with 100 mM L-HSE and 0.5 μM BsHSD to determine the kinetic parameters for substrate and cofactor. The initial reaction rate was measured for L-HSE and NADP^+^ at 6, 7 different concentrations, ranging from 1mM to 300 mM and 20 mM to 2000 mM, respectively. The concentration of L-HSE was varied with NADP^+^ concentration fixed at 2 mM, or the concentration of NADP^+^ was varied with L-HSE concentration fixed at 100 mM. Freshly purified protein samples were used for all enzyme assays and the data from 3 independent experiments were averaged for one measurement value.

 All apparent kinetic parameters were calculated with Origin 9.0 software (OriginLab, USA). The Michaelis-Menten equation *V* = *V*_max_ S/(*K*_m_+S) was used as the reference equation to calculate the apparent parameters from the optimal fitting.

### Size-Exclusion Chromatography Analysis

Size-exclusion chromatography was carried out using a Superdex-200 10/300 GL column on an AKTA basic system (GE Healthcare). A 200 µl protein sample was injected into the column, which was pre-equilibrated with working buffer (20 mM Tris-HCl, 0.1 mM TCEP, and 400 mM NaCl, pH 8.0). The injected protein sample was run through the column at a flow rate of 0.5 mL/min. To determine the void volume, Blue dextran (Sigma-Aldrich, D4772) was used. Next, the mixture of the other 8 standard proteins was run: aprotinin from bovine lung (Sigma-Aldrich, A3886), cytochrome C from equine heart (Sigma-Aldrich, C7150), carbonic anhydrase from bovine erythrocytes (Sigma-Aldrich, C7025), ovalbumin from chicken egg white (Sigma-Aldrich, A8581), alcohol dehydrogenase from yeast (Sigma-Aldrich, A8656), β-amylase from sweet potato (Sigma-Aldrich, A8781), and apoferritin from horse spleen (Sigma-Aldrich, A3630). From all the above elution volume measurements, the standard curve for the molecular mass was established. The elution volume of BsHSD was also measured under conditions similar to those used for the standard proteins.

### Circular Dichroism (CD) Spectroscopy and Thermal Denaturation Analysis

Prior to the CD measurements, the buffer condition for the BsHSD sample was changed (20 mM Tris-HCl pH 8.0, 400 mM NaF, and 0.1 mM TCEP) and a HiPrep 26/10 Desalting column was used. The far-ultraviolet CD spectrum of BsHSD was recorded at 20oC within a cell with 0.1 cm path length using a Jasco J-710 spectropolarimeter (JASCO, USA). Three individual scans were recorded from 190 nm to 260 nm (0.1 nm step resolution, 1 nm bandwidth and 1 s response time). The solvent CD signal was subtracted after summing and averaging 3 spectra. The CD intensity was normalized to the mean residue molar ellipticity. The thermal denaturation experiment was carried out at 222 nm for BsHSD at a concentration of 0.5 mg/ml. The CD intensity was recorded every 30 s as the temperature increased from 26oC to 94oC at a speed of 2oC/min.

### Homology Modeling

The three-dimensional structure was predicted by SWISS-MODEL (http://swissmodel.expasy.org) from the amino acid sequence of BsHSD. The server selected the crystal structure of HSD from *Mycolicibacterium hassiacum* (PDB - 6DZS; not published) as a template, on which the structure of BsHSD was modeled.

## Results

### BsHSD Expression and Purification

**Fig. 2:**
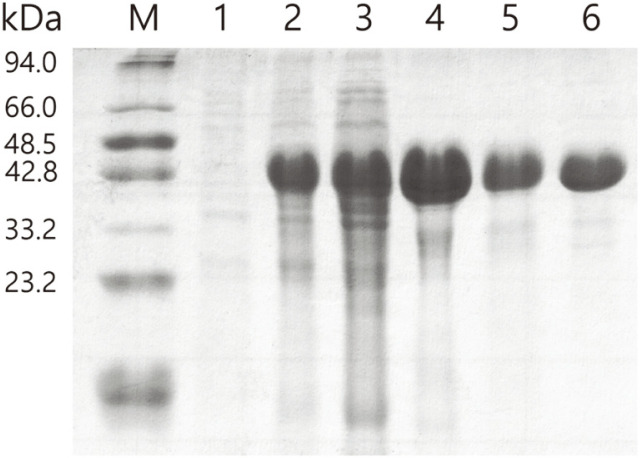
Purification of recombinant BsHSD. SDS-PAGE for the samplings in the steps of culturing and purification. Lane M: protein marker; Lane 1: before IPTG addition; Lane 2: overnight, after IPTG addition; Lane 3: supernatant after cell lysis; Lane 4: elution from HistrapFF column; Lane5: after Superdex-200 column; and Lane 6: final concentrated sample.

Expression of the recombinant BsHSD in *E. coli* at 37°C resulted mostly into inclusion bodies. However, we found that it can be expressed in soluble form at a lower temperature of 25°C. The band for expressed BsHSD appeared between two markers, 48.5 kDa to 42.8 kDa, which is in good agreement with its calculated molecular mass of 48.3 kDa. The expressed recombinant BsHSD was purified through a series of chromatography columns. The final purified sample showed remarkably high homogeneity, which was assessed with SDS-PAGE (Fig. 2), and it was applied to the subsequent molecular and enzymatic analyses.

### Optimal Enzymatic Condition and Cofactor Specificity

**Fig. 3:**
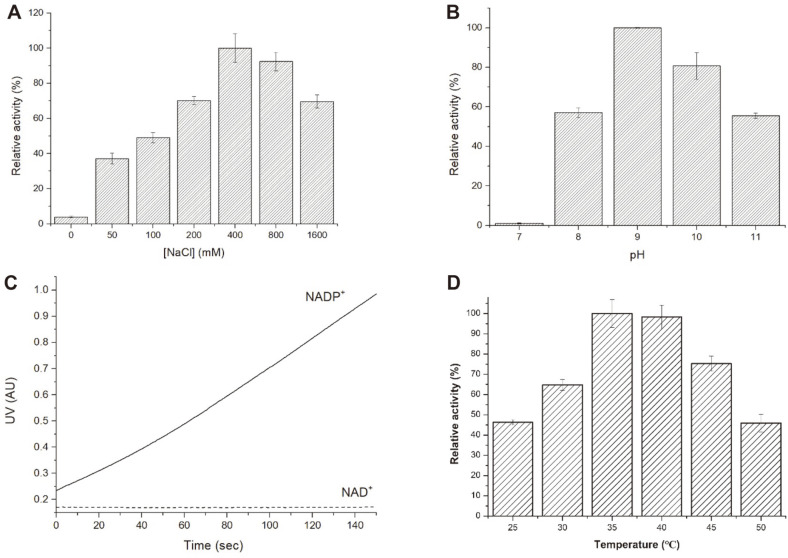
Optimum conditions for BsHSD. The specific activity was measured at varying points of parameters to inspect the optimum condition. (**A**) Effect of NaCl concentration (**B**) pH dependence (**C**) Cofactor specificity (**D**) Temperature dependence of the enzymatic activity.

To establish the optimal salt concentration and pH for the L-HSE oxidation reaction catalyzed by BsHSD, we measured the catalytic activity at varied NaCl concentrations and pH, respectively. First, we tested 7 different NaCl concentrations ranging from 0 to 1.6 M at pH 9.5 (Fig. 3A). The results clearly showed that the presence of NaCl is greatly beneficial for the enzyme activity and even with only 50 mM NaCl, the activity was about 11 times greater than the activity without NaCl. As the NaCl concentration was doubled from 50 mM to 100 mM, and then sequentially to 1,600 mM, the activity increased to reach its maximum at 400 mM NaCl with a specific activity of 1.48 ± 0.12 μmol·min^-1^·mg^-1^. Thus, all the subsequent assays were carried out with the 100 mM Tris-HCl buffer containing 400 mM NaCl. Next, we investigated the pH dependence of L-HSE oxidation by measuring the activity at 5 different pHs (7.0, 8.0, 9.0, 10.0, and 11.0) (Fig. 3B). BsHSD was maximally active for L-HSE oxidation at pH 9.0 with a specific activity of 2.01 ± 0.01 μmol·min^-1^·mg^-1^ and was the least active at pH 7.0 with only 1.1 % of the maximum activity. We also examined the cofactor specificity of L-HSE oxidation for NAD^+^ and NADP^+^. BsHSD exhibited absolute preference for NADP^+^ and the activity with NAD^+^ was not detectable at all (Fig. 3C). We also carried out the homoserine oxidation assay at different reaction temperatures ranging from 25°C to 50°C to investigate the temperature dependence of the enzyme activity. As the temperature increased, the specific activity also increased to 35°C and then gradually decreased to 40°C (Fig. 3D).

### Apparent Kinetic Parameters

**Fig. 4:**
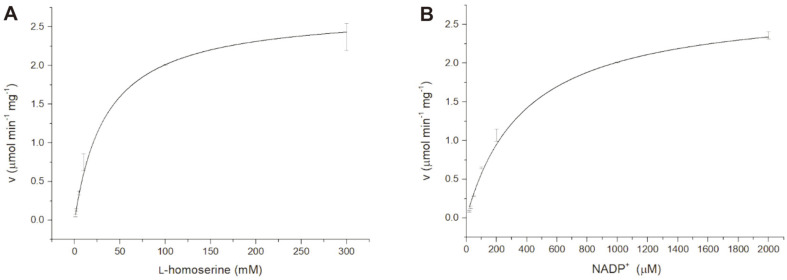
Michaelis-Menten kinetics of BsHSD. The specific activity was measured at varying concentration points of substrate or cofactor to determine the kinetic parameters. (**A**) _L_-homoserine concentration was varied with saturated concentration of NADP^+^. (**B**) NADP^+^ concentration was varied with saturated concentration of _L_-homoserine.

To determine the kinetic parameters for the oxidation of _L_-homoserine by BsHSD, we measured the initial reaction rates at various concentrations of _L_-homoserine with NADP^+^ saturation or conversely at various concentrations of NADP^+^ with _L_-homoserine saturation (Fig. 4). The *K*_m_ values for _L_-homoserine and NADP^+^ were 35.08 ± 2.91 mM and 0.39 ± 0.05 mM (Table 1), respectively, and the *V*_max_ values were quite similar at 2.72 ± 0.06 μmol·min^-1^·mg^-1^ and 2.79 ± 0.11 μmol·min^-1^·mg^-1^ respectively, with the *k*_cat_/*K*_m_ value as 0.03 ± 0.01 sec-1 mM^-1^, indicating that the binding of _L_-homoserine is the rate limiting factor for _L_-homoserine oxidation.

### Size-Exclusion Chromatography Analysis

**Fig. 5:**
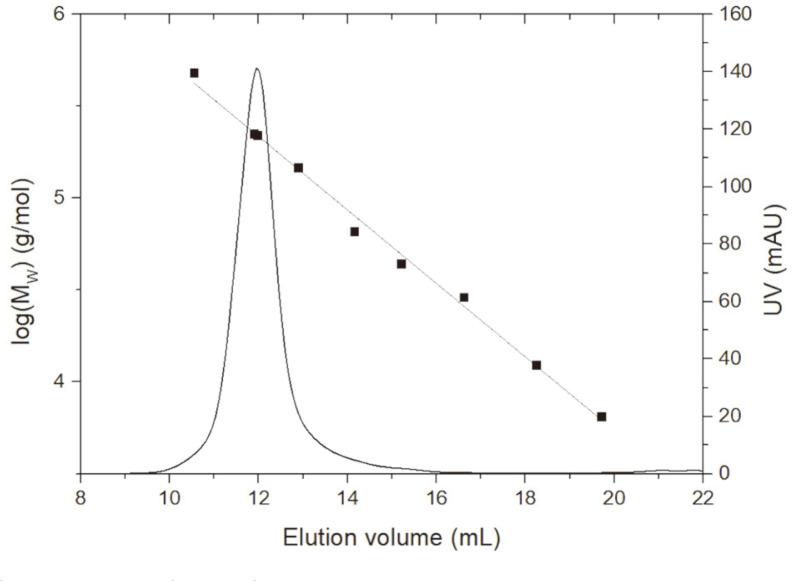
Size-exclusion chromatography analysis. The molecular mass of BsHSD was measured in size-exclusion column chromatography to investigate the oligomeric state in solution. Filled squares are 8 different standard proteins. The peak corresponds to BsHSD eluted at 12.0 ml.

To examine the oligomeric states of BsHSD, we carried out size-exclusion chromatography. The molecular mass was estimated by comparing the elution volume of BsHSD with those of the standard proteins (Fig. 5). The BsHSD peak emerged at 12.0 ml of elution volume and the molecular mass was calculated to be 221 kDa from the standard curve, which was 4.6 times greater than the actual molecular mass of monomeric BsHSD (48.3 kDa). This result indicates that BsHSD exists as a tetramer in solution.

### Circular Dichroism Spectroscopy and Thermal Denaturation

**Fig. 6:**
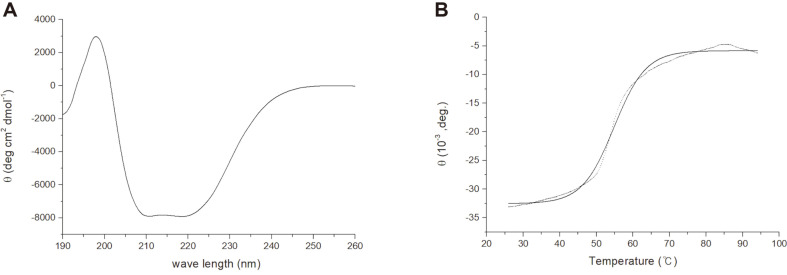
Circular dichroism analysis. The fold integrity and the secondary structure composition was checked from the wavelength scan, and the thermal denaturation experiment was carried out to investigate the thermal stability. (**A**) Wavelength scan (**B**) Thermal denaturation at 222 nm. The dotted line represents raw measurements, and the solid line represents their sigmoidal fitting.

The CD spectra were recorded for the wavelength scan from 190 nm to 260 nm at 25°C (Fig. 6A), and it exhibited a typical pattern for α/β proteins. The content of the secondary structure elements was estimated by BeStSel [13], and it showed 16.7% for helices, 25.0% for strands, 14.3% for turns, and 43.9% for the others. This is largely consistent with the previous reports of the crystal structures of HSDs from other organisms which revealed a common α/β structure including a Rossman fold [2-6]. In addition, this result suggests the structural integrity of the purified BsHSD sample. Next, thermal denaturation experiments were performed using CD spectroscopy to investigate the thermal stability of BsHSD. The spectrum was measured every 30 s at 222 nm, raising the sample temperature from 26°C to 94°C and a total of 336 data points were collected. The melting temperature (Tm) was determined from the sigmoid fitting of these data at 54.8 ± 0.1oC, which fell within the range for mesophilic proteins (20°C to 60°C) [14, 15].

### Predicted Model for Three-Dimensional Structure

**Fig. 7:**
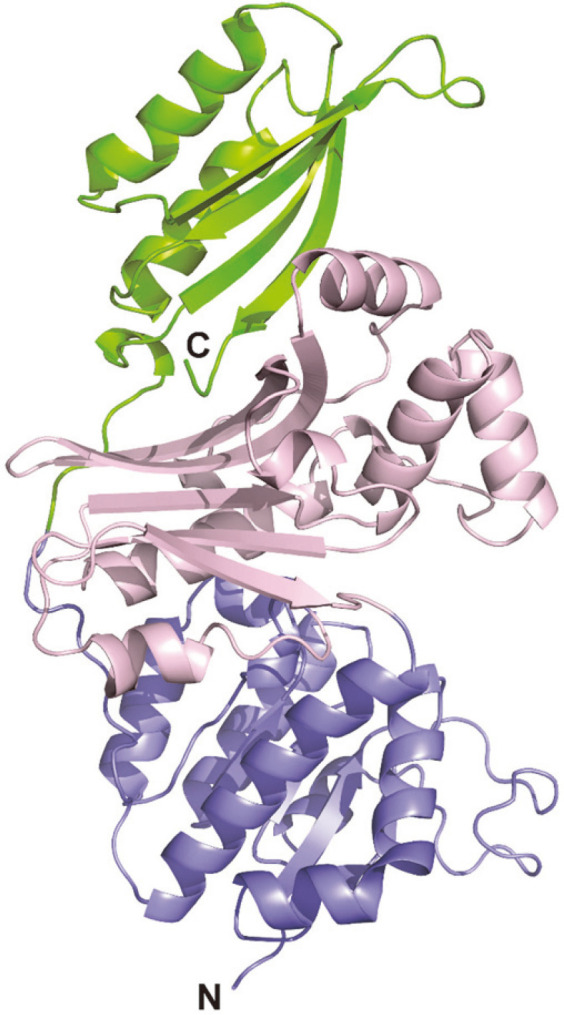
Predicted model for three-dimensional structure of BsHSD. The N-terminal nucleotide-binding domain is shown in blue, the central substrate-binding domain in pink, and the C-terminal ACT domain in green.

The three-dimensional structure of BsHSD was predicted using the SWISS-MODEL server (http://swissmodel.expasy.org) based on the crystal structure of HSD from *M. hassiacum* (PDB - 6DZS; MhHSD, hereafter; not published) as a homology model that was algorithmically selected by the server. MhHSD showed 40.3% sequence identity with BsHSD on an alignment covering 430 residues with several short gaps. The model revealed that BsHSD consists of 3 domains: the N-terminal nucleotide-binding domain of a varied Rossman fold, a central substrate-binding domain, and a C-terminal ACT domain (acronym for aspartate kinase, chorismate mutase and TyrA (prephenate dehydrogenase)) of a ferredoxin-like fold (Fig. 7) [5]. The nucleotide-binding domain and the substrate-binding domain are commonly found in all HSDs from any organism, but the C-terminal ACT domain is an additional regulatory domain that is present in only a subset of HSDs [2-6].

## Discussion

In this study, BsHSD, was recombinantly expressed, purified and characterized for its molecular and enzymatic features. We investigated the optimal pH, temperature, and NaCl concentration for the enzyme activity and determined the kinetic parameters for the oxidation of _L_-homoserine. Next, we also examined the oligomeric state of BsHSD in solution through size-exclusion chromatography, and its thermal stability by CD spectroscopic thermal denaturation test. Finally, we built a predicted model for three-dimensional structure of BsHSD. Based on all these analyses, a few novel features of BsHSD were revealed. In the preliminary enzyme assays for the optimization of the reaction conditions, we observed that the enzyme activity was greatly enhanced (almost 11 times) by the addition of NaCl even at very low concentration (50 mM) and the activity reached its maximum at 400 mM of NaCl concentration. In this regard, it should be noted that BaHSD showed the tendency to aggregate over time, as assessed by the appearance of the peak at void volume in the absence of NaCl and the removal of this aggregation almost completely by the addition of 400 mM NaCl, as shown by the size-exclusion chromatography data. Therefore, it seems that the activity enhancement by NaCl may have resulted at least in part because of preventing aggregation.

Since the dimer is a typical oligomeric state for the previously reported HSDs from other organisms [2-6], the tetramer formation of BsHSD was an unexpected observation. For example, HSD from *S. aureus* was shown to exist as a dimer in solution as examined by size-exclusion chromatography [5], which was consistent with its crystal structure revealing the dimeric assembly. Moreover, HSDs from *S. cerevisiae*, *T. thermophilus*, *P. horikoshii*, and *S. tokodaii* also showed the same dimeric assembly in the crystal lattice [2-4,6], even though their oligomeric state was not investigated in solution. These previous crystal structures of HSDs commonly showed that the dimeric interface was contributed by both the substrate-binding domain and the nucleotide-binding domain. Thus, to form the tetramer, BsHSD needs another type of dimeric interaction in addition to the above conserved one. In this regard, it is noteworthy that BsHSD contains the additional C-terminal ACT domain, which is not present in all of the above dimeric HSDs, with the exception of HSD from *S. aureus*. More importantly, the ACT domain was shown to be responsible for the dimerization of the many other ACT domain-containing enzymes such as PGDH, AK, AHAS, and TD, which are all HSDs involved in amino acid biosynthesis processes [7-12]. An ACT domain adopts a ferredoxin-like fold of βαββαβ which has a four-stranded β-sheet with 2 α-helices flanked on one side. In the crystal structures of these ACT domain-containing enzymes, 2 ACT domains accomplish dimerization in a conserved way with 2 four-stranded β-sheets joined edge-to-edge into a single eight-stranded sheet. Therefore, it is very tempting to speculate that the additional dimeric interface responsible for the tetramer formation of BsHSD must be established by its C-terminal ACT domain. Subsequent structural analysis will clearly reveal the details of the novel tetrameric assembly of BsHSD.

Conclusively, the molecular and biochemical features of BsHSD were investigated in this study. Given the importance of HSD as a key enzyme in the aspartate pathway and also as a target for medical and industrial applications, this study will add to the knowledge on the biochemistry of amino acid metabolism and lay the foundation for future efforts in corresponding applications.

## Figures and Tables

**Table 1:** Kinetic parameters.

Substrate	*V_max_* (μmol min^-1^ mg^-1^)	*K_m_* (mM)	*k_cat_* (sec^-1^)	*k_cat_/K_m_* (sec^-1^ mM^-1^)
_L_-homoserine	2.72±0.06	35.08±2.91	1.07±0.02	0.03±0.01
NADP^+^	2.79±0.11	0.39±0.05	1.10±0.04	2.86±0.91
